# Early hyperoxia and 28-day mortality in patients on venoarterial ECMO support for refractory cardiogenic shock: a bicenter retrospective propensity score-weighted analysis

**DOI:** 10.1186/s13054-022-04133-7

**Published:** 2022-08-26

**Authors:** Mouhamed Djahoum Moussa, Christophe Beyls, Antoine Lamer, Stefan Roksic, Francis Juthier, Guillaume Leroy, Vincent Petitgand, Natacha Rousse, Christophe Decoene, Céline Dupré, Thierry Caus, Pierre Huette, Mathieu Guilbart, Pierre-Grégoire Guinot, Patricia Besserve, Yazine Mahjoub, Hervé Dupont, Emmanuel Robin, Jonathan Meynier, André Vincentelli, Osama Abou-Arab

**Affiliations:** 1 Pôle d’Anesthésie-Réanimation, Lille Hospital University, 59000 Lille, France; 2 Anesthesia and Critical Care Medicine Department, Amiens University Medical Center, 1 rue du Professeur Christian Cabrol, 80054 Amiens, France; 3 CHU Lille, ULR 2694-METRICS : Évaluation des Technologies de Santé Et des Pratiques Médicales, 59000 Lille, France; 4 Cardiac Surgery, Lille Hospital University, 59000 Lille, France; 5 Cardiac Surgery, Amiens University Medical Center, 80054 Amiens, France; 6 Department of Anesthesiology and Critical Care Medicine, Dijon University Hospital, 21000 Dijon, France; 7 Department of Biostatistics, Amiens Picardy University Hospital, 80054 Amiens, France

**Keywords:** Cardiogenic shock, ECMO, Hyperoxia, Mortality

## Abstract

**Background:**

The mortality rate for a patient with a refractory cardiogenic shock on venoarterial (VA) extracorporeal membrane oxygenation (ECMO) remains high, and hyperoxia might worsen this prognosis. The objective of the present study was to evaluate the association between hyperoxia and 28-day mortality in this setting.

**Methods:**

We conducted a retrospective bicenter study in two French academic centers. The study population comprised adult patients admitted for refractory cardiogenic shock. The following arterial partial pressure of oxygen (PaO_2_) variables were recorded for 48 h following admission: the absolute peak PaO_2_ (the single highest value measured during the 48 h), the mean daily peak PaO_2_ (the mean of each day’s peak values), the overall mean PaO_2_ (the mean of all values over 48 h), and the severity of hyperoxia (mild: PaO_2_ < 200 mmHg, moderate: PaO_2_ = 200–299 mmHg, severe: PaO_2_ ≥ 300 mmHg). The main outcome was the 28-day all-cause mortality. Inverse probability weighting (IPW) derived from propensity scores was used to reduce imbalances in baseline characteristics.

**Results:**

From January 2013 to January 2020, 430 patients were included and assessed. The 28-day mortality rate was 43%. The mean daily peak, absolute peak, and overall mean PaO_2_ values were significantly higher in non-survivors than in survivors. In a multivariate logistic regression analysis, the mean daily peak PaO_2_, absolute peak PaO_2_, and overall mean PaO_2_ were independent predictors of 28-day mortality (adjusted odds ratio [95% confidence interval per 10 mmHg increment: 2.65 [1.79–6.07], 2.36 [1.67–4.82], and 2.85 [1.12–7.37], respectively). After IPW, high level of oxygen remained significantly associated with 28-day mortality (OR = 1.41 [1.01–2.08]; *P* = 0.041).

**Conclusions:**

High oxygen levels were associated with 28-day mortality in patients on VA-ECMO support for refractory cardiogenic shock. Our results confirm the need for large randomized controlled trials on this topic.

**Supplementary Information:**

The online version contains supplementary material available at 10.1186/s13054-022-04133-7.

## Background

Venoarterial extracorporeal membrane oxygenation (VA-ECMO) is recommended as a rescue therapy for ensuring organ perfusion and oxygen delivery in cases of refractory cardiogenic shock (CS) [1]. The survival rate for CS patients on VA-ECMO varies from one study to another; the largest study to date (an analysis of more than 2000 patients documented in the Extracorporeal Life Support Organization database) found a value of 40% [2].

A large number of factors can influence the mortality of CS patients on VA-ECMO support, including patient characteristics, the etiology of the CS, the center’s level of experience, and adverse bleeding/thrombotic events [3–6]

Hyperoxia is a modifiable factor that reportedly worsens outcomes in several critical illnesses and might contribute to the elevated mortality rates observed in patients with refractory CS [7]. The hypothesis is that hyperoxia might increase oxidative stress by triggering enzymatic pathways that result in greater production of free radicals and reactive oxygen species (ROS) [8]. This elevation in ROS generation might promote neutrophil activation, which might in turn lead to an inappropriate inflammatory response [9], 10. Lung injury associated with hyperoxia is well described with loss in hypoxic pulmonary vasoconstriction and formation of atelectasis [11]. Therapeutics issues from animal models are described to protect the lung against hyperoxia injuries as the use of luciferase acting like an antioxidant reduces the production of ROS [12]. However, clinical reports on oxygen management during VA-ECMO support for CS are scarce, and the data on hyperoxia in this setting are contradictory. An analysis of the Extracorporeal Life Support Organization database did not find an association between hyperoxia and VA-ECMO [13]. However, the analysis included patients with refractory CS and those with refractory cardiac arrest, which means that the data must be interpreted with caution [13], 14. Although hyperoxia appears to be associated with mortality during venovenous ECMO [13], the data on VA-ECMO in CS are inconsistent [15].

We hypothesized that in a homogenous population of patients with low cardiac output and severe organ oxygen deprivation, hyperoxia might compromise the function of organs with ischemia perfusion injuries during initial patient management with VA-ECMO.

The objective of the study was therefore to evaluate the putative association between early hyperoxia and 28-day mortality in patients on VA-ECMO support.

## Methods

### Setting and ethics

This retrospective, multicenter study was performed in three intensive care units (ICUs) at two university medical centers (Unité de Réanimation Cardio-Vasculaire, Institut Cœur-Poumon, Lille University Medical Center, Lille, France; Pôle des Réanimations, Hôpital Salengro, Lille University Medical Center, Lille, France; Unité de Réanimation Cardio-Thoracique, Vasculaire et Respiratoire, Amiens University Medical Center, Amiens, France). The study was approved by the investigational review board at the French Society of Anesthesia, Intensive Care and Perioperative Medicine (Paris, France) on January 17, 2022 (reference: CERAR IRB 00,010,254-2022-009). In accordance with French legislation, all datasets used in the present study were registered with the French National Data Protection Commission (*Commission nationale de l'informatique et des libertés* (Paris, France; methodology MR-004; reference: 2208336v0 for Amiens University Medical Center, and DEC2015-14 for Lille University Medical Center) [16].

### Study population

Consecutive adult patients on VA-ECMO support for refractory CS rated as Interagency Registry for Mechanically Assisted Circulatory Support profile 1 or 2 (“crash and burn” and “progressive decline on inotropic support,” respectively) or Society for Cardiovascular Angiography and Interventions stage D or E (“deteriorating” and “extremis,” respectfully) between January 2013 until January 2020 were considered for inclusion [17, 18].

ECMO initiated exclusively for high-risk percutaneous coronary intervention, cardiopulmonary resuscitation, or an intraoperative procedure was not included in the study. The formal exclusion criteria were age under 18, a moribund patient (death within 48 h of initiating VA-ECMO), missing data for PaO_2_, uncertainty as to whether a blood sample came from the right arterial or ulnar arteries (in cases of femoral artery cannulation), a second ECMO run in the same patient, central cannulation, and right ventricle to pulmonary artery ECMO.

### Data collection

Clinical data and outcome data were gathered from paper-based or electronic medical records [Sillage (SIB, Rennes, France) and IntelliSpace Critical Care and Anesthesia (Philips Healthcare, Koninklijke Philips N.V., the Netherlands) for the Lille centers and Centricity Critical Care (formerly known as Clinisoft) software (GE Healthcare, Barrington, IL) for the Amiens center. Laboratory information was collected from devoted software applications [Molis^®^ (CompuGroup Medical, Koblenz, Germany) in the Lille centers and Clinisoft (GE Healthcare, Barrington, IL) in the Amiens center]. We collected anthropometric data, a detailed medical history, the Simplified Acute Physiologic Score (SAPS II), the baseline arterial blood lactate level, and other laboratory variables. We also collected all relevant information concerning ECMO management, including the indication for ECMO support, the cannulation site, the duration of cannulation, the type of device, the ECMO flow, and the occurrence of any ECMO-related complications.

### Blood samples and laboratory analysis

Arterial blood samples were drawn into a 3-mL preheparinized syringe (BD Preset^™^, Plymouth, UK) from arterial lines positioned in the right radial artery. The samples were processed within minutes of collection on a point-of-care analyzer or sent to a central laboratory using an automatized pneumatic tube transportation system that shortened the laboratory delivery time to a few minutes. PaO_2_ was measured using an ABL90 FLEX or ABL800 FLEX blood gas analyzer (Radiometer^®^ Medical ApS, Brønshøj, Denmark) or a GEM^®^Premier 4000 blood gas analyzer (Instrumentation Laboratory, Werfen, Bedford, MA, USA) depending on the center.

### Management of VA-ECMO

The femoral artery or the right subclavian artery was cannulated percutaneously or with a semi-Seldinger approach. The femoral vein was cannulated by trained cardiovascular or thoracic surgeons. During the study period, three ECMO systems were in use: a Maquet ECMO system, comprising a Rotaflow centrifugal pump-based system and a Cardiohelp with a disposable 5.0/7.0HLS Set Advanced (Getinge AB, Göteborg, Sweden); a LivaNova system (LivaNova, Saluggia, Italy) with a revolution pump head; and a Eurosets system (Eurosets Srl, Medolla, Italy)].

Anticoagulation was initiated with a 100 IU/kg bolus of unfractionated heparin before cannulation for patients not on cardiopulmonary bypass prior to ECMO support, followed by a continuous infusion. The target was an anti-FXa level of 0.2–0.4 IU ml^−1^ in the Lille and Amiens centers.

The pump flow was adjusted to target a mean arterial pressure > 60 mmHg, SvO_2_ > 65% or ScVO_2_ > 70%, and aortic valve opening. Weaning was considered for recovery when the cardiac output was acceptable after reducing the ECMO flow and inotropic support to the minimum level.

It should be noted that PaO_2_ was adjusted at the discretion of the attending physician—primarily by changing the ECMO system’s fraction of inspired oxygen (FiO_2_) via an oxygen–air blender (Sechrist Industries, Anaheim, CA). The FiO_2_ on the ventilator was set to the minimum value and was modified as a function of the arterial oxygen saturation (SaO_2_) measured in the right upper limb arteries (for femoral cannulation) or the left upper limb arteries (for right subclavian arterial cannulation) or the cerebral near-infrared spectrometry index values when the SaO_2_ or peripheral oxygen saturation (SpO_2_) values were unavailable.

### Definition of oxygen parameters

The following PaO_2_ values (in mmHg) were recorded or calculated over the first 48 h following admission to the ICU: (i) the three through PaO_2_ values on admission (Day 0), Day 1, and Day 2; (ii) the three peak PaO_2_ values on admission, Day 1, and Day 2; (iii) the mean daily peak PaO_2_ (mmHg), calculated as the mean of the daily peak values on admission, Day 1, and Day 2; (iv) the mean daily through PaO_2_, calculated as the mean of the daily through values; (v) the absolute peak PaO_2_, i.e., the highest peak PaO_2_ value between admission and Day 2; (vi) the overall mean PaO_2_, i.e., the mean of all PaO_2_ values between admission and Day 2; and (vi) the severity of hyperoxia, graded with reference to the overall mean PaO_2_ (mild: < 200 mmHg; moderate: 200–299 mmHg; severe: ≥ 300 mmHg).

### Study endpoint

The primary study endpoint was 28-day all-cause mortality, as determined from the patient’s electronic medical records and the French national death registry (*Institut national des statistiques et des études économiques*, Paris, France) [19]. None of the study participants was lost to follow-up, and status at 28 days could be documented in all cases.

### Statistical analysis

Data were presented as the mean ± standard deviation or the frequency (percentage), as appropriate. Non-survivors were compared with 28-day survivors using Student’s t test, a chi-squared test, or Fisher’s exact test, as appropriate. To assess the effects of ECMO flow rate and hemoglobin on PaO_2_, we used a multiple linear regression. Binary logistic regression analyses with 28-day mortality as the dependent variable were used to estimate the respective univariate associations with the absolute peak PaO_2_, mean daily peak PaO_2_, the overall mean PaO_2_, and the hyperoxia range. Unadjusted and adjusted odds ratios (ORs) with their 95% confidence interval (CI) were estimated from the binary logistic regression for a 10-point increment in PaO_2_-derived parameters. The results were adjusted for age, hypertension, the indication for VA-ECMO support, the SAPS II, and the arterial blood lactate on admission. Kaplan–Meier estimates were used for time-to-event analyses. Sensitivity analyses were performed by excluding patients with femoro-axillary ECMO and by using the OR as a measure of the effect size.

Missing data have been imputed for the following stages using predictive mean matching imputation (pmm). Five imputation data sets were produced. Missing data have been analyzed as missing-not-at-random according to the Rubin rule [20]. Prognostic variables related to 28-day mortality at the 20% in univariate analysis were included in the propensity score (regardless of their differences between the two groups): age, gender, hypertension, eGFR, diabetes, coronary disease, SAPS II, arterial lactate on admission, and etiology of refractory cardiogenic shock. For each patient, the probability to show hyperoxia has been estimated using logistic regression. For ATE (average treatment effect on the entire population) analysis, weights have been attributed to each patient of the hyperoxia (overall mean PaO2 > 150 mmHg) and no hyperoxia (overall mean PaO_2_ ≤ 150 mmHg) groups, making the two groups similar for the variables in the propensity score [21]. These weights were calculated using the stabilized inverse probability of treatment weighting (SIPTW). Balance (standardized mean differences lower than 10%) was checked for each variable. ORs were estimated before and after weighting on the propensity score using logistic regression. All tests were two-sided, and the threshold for statistical significance was set to *p* < 0.05. Statistical analysis was performed with R studio software for macOS (version 2021.09.1 + 372) and its «dplyr», «ggplot2», «survminer», «survival», « hrbrthemes», «tableone», « ggeffects», « WeightIt», « cobalt», « compareGroups», « mice» and « epiR» and “reshape2” packages.

## Results

### Study population

**Fig. 1:**
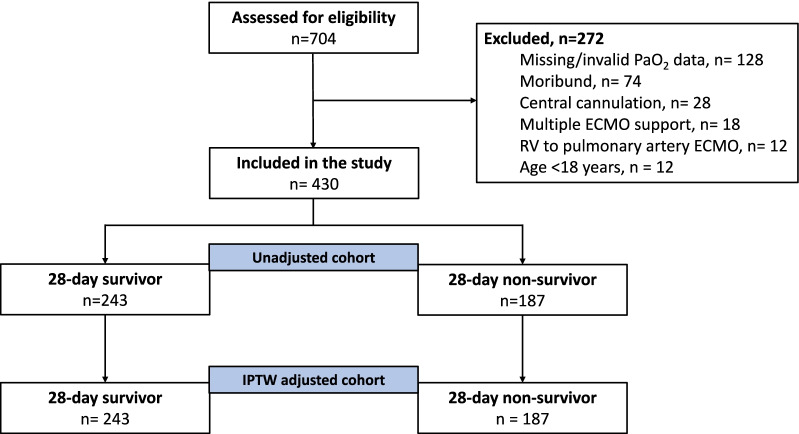
Flowchart. ECMO: extracorporeal membrane oxygenation; RV: right ventricle; IPTW: inverse probability of treatment weighting

From January 2013 to January 2020, a total of 704 patients received VA-ECMO support; 272 did not meet the inclusion criteria, and so the final analysis comprised 430 patients (Fig. 1).

### Baseline characteristics

**Fig. 2:**
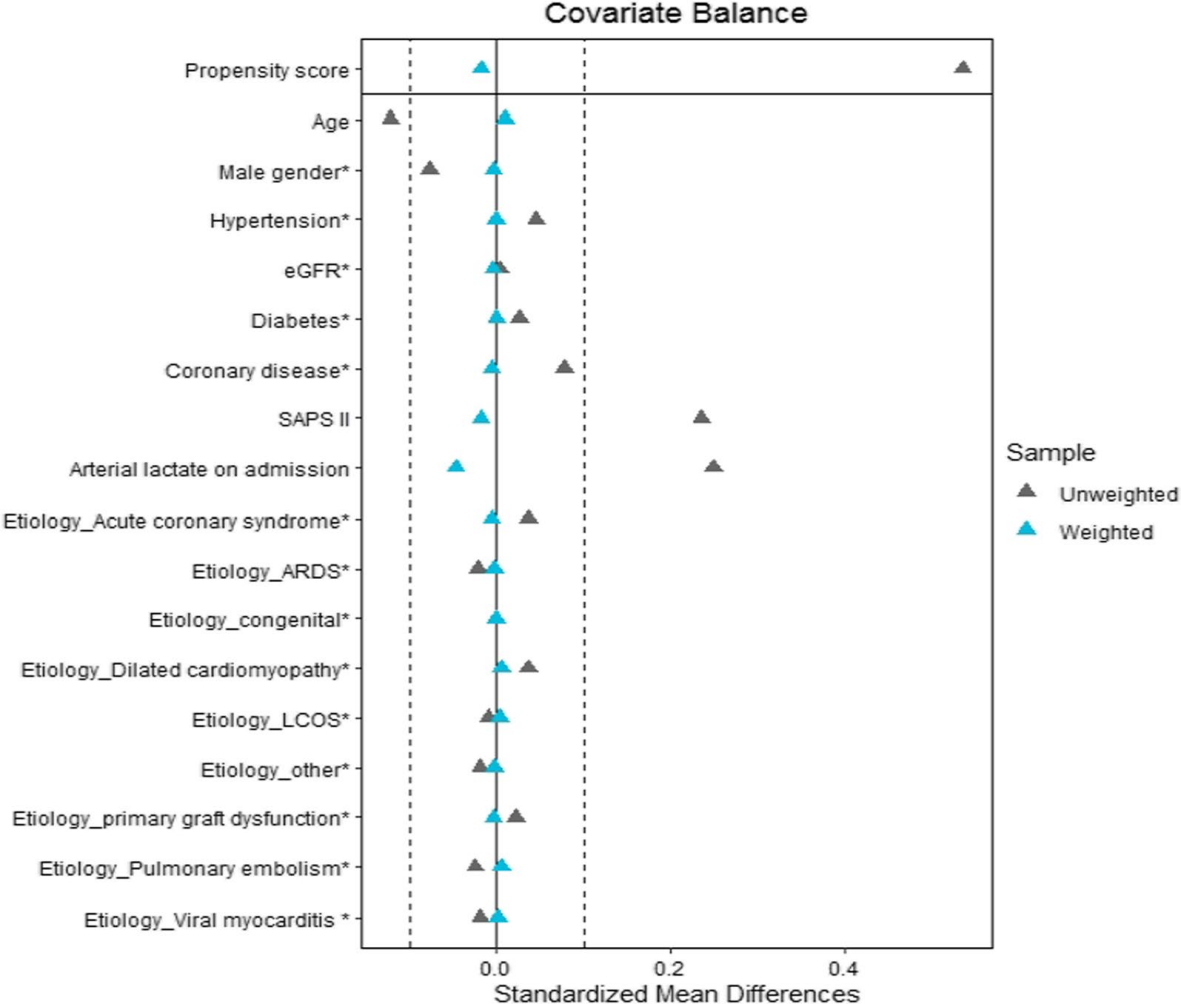
Love plots for standardized mean differences comparing covariate values before (gray triangle) and after (blue triangle) propensity score weighting for the assessment of 28-day mortality. LCOS: low cardiac output syndrome; SAPS II: Simplified Acute Physiology Score II; eGFR: estimated glomerular filtration rate; ARDS: acute respiratory distress syndrome

Relative to survivors, non-survivors were significantly older and more likely to have a history of hypertension (Table 1). The non-survivors had a significantly greater SAPS II and arterial blood lactate level on admission. Before weighting, the prevalence of acute coronary syndrome and low cardiac output syndrome was significantly higher in non-survivors. The 28-day mortality rate was 43%. After weighting, the mean standard differences were inferior to 15% for all baseline characteristics as presented in Table 1 and in the love plot (Fig. 2).

**Table 1:** Baseline characteristics of patients (28-day survivors vs. non-survivors) on venoarterial ECMO support before and after weighting

	Before weighting			After weighting		
Variable	Survivors (*n* = 243)	Non-survivors (*n* = 187)	SMD	Survivors (*n* = 243)	Non-survivors (*n* = 187)	SMD
Age; *years*	53 ± 14	59 ± 13	0.122	58 ± 10	58 ± 10	0.010
Male sex; *n (%)*	155 (64)	134 (72)	0.165	168 (69)	127 (68)	0.008
BMI	27.6 ± 6.1	28.0 ± 5.8	0.084	27.9 ± 6	27.9 ± 7	0.046
*Medical history; n (%)*						
Hypertension	102 (42)	102 (55)	0.090	119 (49)	92 (49)	0.001
eGFR < 80 ml min^−1^ m^−2^	103 (43)	98 (53)	0.007	116 (48)	90 (49)	0.008
Diabetes	54 (22)	54 (29)	0.059	61 (25)	47 (25)	< 0.001
Stroke	14 (6)	13 (7)	0.009	17 (7)	13 (7)	0.003
Coronary disease	99 (41)	90 (49)	0.156	109 (45)	84 (45)	0.011
COPD/asthma	22 (9)	23 (12)	0.132	27 (11)	23 (12)	0.109
Dilated cardiomyopathy	31 (13)	19 (10)	0.09	29 (12)	21 (11)	0.04
Valvular heart disease	50 (21)	46 (25)	0.173	56 (23)	43 (23)	0.149
SAPS II	54 [38–71]	57 [45–74]	0.235	56 [43–73]	54 [43–72]	0.018
Arterial lactate on admission; *mmol l*^*−1*^	6 ± 4	8 ± 5	0.249	5 ± 4	5 ± 4	0.044
*Etiology of refractory cardiogenic shock; n (%)*						
LCOS	63 (26)	54 (29)	0.265	66 (27)	51 (27)	0.038
Primary graft dysfunction	16 (7)	4 (2)		12 (5)	9 (5)	
Acute coronary syndrome	63 (26)	58 (31)		68 (28)	52 (28)	
Dilated cardiomyopathy	37 (15)	16 (9)		27 (11)	22 (12)	
Viral myocarditis	13 (5)	4 (2)		10 (4)	7 (4)	
Pulmonary embolism	13 (5)	9 (5)		12 (5)	11 (6)	
Congenital	3 (1)	1 (1)		3 (1)	2 (1)	
ARDS	5 (2)	5 (3)		5 (2)	4 (2)	
Other	30 (12)	30 (20)		39 (16)	30(16)	

BMI, body mass index; eGFR, estimated glomerular filtration rate; COPD, chronic obstructive pulmonary disease; ECMO, extracorporeal membrane oxygenation; SAPS II, Simplified Acute Physiology Score II; LCOS, low cardiac output syndrome; ARDS, acute respiratory distress syndrome; SMD: standardized mean differences

### Comparisons of oxygen levels in survivors versus non-survivors

Survivors and non-survivors did not differ significantly with regard to the daily through PaO_2_ from ICU admission to Day 2 (Table 2). The daily peak PaO_2_ was significantly higher in non-survivors than in survivors on admission (mean ± SD: 258 ± 111 vs. 228 ± 100 mmHg, respectively; *P* = 0.007) and on Day 1 (and 237 ± 93 vs. 208 ± 87 mmHg, respectively; *P* = 0.001) but not on Day 2. The non-survivors and survivors did not differ significantly with regard to the daily through PaO_2_ (94 ± 44 vs. 92 ± 37 mmHg, respectively; *P* = 0.567). The mean daily peak PaO_2_, the absolute peak PaO_2_, and the overall mean PaO_2_ were significantly higher in non-survivors than in survivors (respectively 221 ± 66 vs. 202 ± 51 mmHg; *P* = 0.004, 296 ± 100 vs. 265 ± 90 mmHg; *P* = 0.002 and 158 ± 42 vs. 147 ± 39 mmHg; *P* = 0.011). The prevalence of moderate and severe hyperoxia was significantly higher in non-survivors than in survivors.

**Table 2:** Daily peak, daily through, and mean PaO_2_ values in 28-day survivors and non-survivors

Variables	Survivors (*n *= 243)	Non-survivors (*n* = 187)	*P* value
*Inspired oxygen fraction (%)*			
ICU admission	0.5 ± 0.1	0.7 ± 0.2	< 0.001
Day 1	0.5 ± 0.1	0.7 ± 0.2	< 0.001
Day 2	0.5 ± 0.1	0.6 ± 0.2	< 0.001
*Daily through PaO*_*2*_*; mmHg*			
ICU admission	108 ± 68	109 ± 85	0.844
Day 1	84 ± 37	86 ± 48	
Day 2	86 ± 34	94 ± 49	0.053
*Daily peak PaO*_*2*_*; mmHg*			
ICU admission	228 ± 100	258 ± 111	0.007
Day 1	208 ± 87	237 ± 93	0.001
Day 2	183 ± 74	191 ± 83	0.369
Mean daily peak PaO_2_; *mmHg* ^a^	202 ± 61	221 ± 66	0.004
Mean daily through PaO_2_; *mmHg* ^b^	92 ± 37	94 ± 44	0.567
Absolute peak PaO_2_ over 48 h; *mmHg*	265 ± 90	296 ± 100	0.002
*PaO*_*2*_* range; n (%)*			
< 200 mmHg	152 (63)	30 (40)	0.010
200 – 299 mmHg	74 (30)	76 (41)	
≥ 300 mmHg	17 (7)	19 (10)	
Overall mean PaO_2_; *mmHg* ^c^	147 ± 39	158 ± 42	0.011

^a^:The mean daily peak PaO_2_ is the mean of the three daily peak PaO_2_ values (measured on admission (Day 0), Day 1, and Day 2). ^b^: The mean daily through PaO_2_ is the mean of the three of the daily through PaO_2_ values (measured on admission, Day 1, and Day 2). ^c^: The overall mean PaO_2_ is the mean of all PaO_2_ values measured between admission and Day 2. ICU: intensive care unit

### Influence of ECMO flow rate and hemoglobin on PaO_2_ level at admission

In a multiple regression model, hemoglobin was significantly associated with PaO_2_ with an estimated regression coefficient at − 10.5 g dl^−1^ (95% CI − 16.3 to − 4.8) per 1 mmHg increase in PaO_2_ (*P* < 0.001). ECMO flow rate was not associated with PaO_2_ at admission with an estimated regression coefficient at 4.5 l min^−1^ (95% CI − 9.1 to 18.2) per 1 mmHg increase in PaO_2_ (*P* = 0.51). The association between hemoglobin, ECMO flow rate, and PaO_2_ is presented in Additional file 1: Fig. S1.

### Univariate and multivariate analyses

In a univariate logistic regression, the mean daily peak PaO_2_, the absolute peak PaO_2_, the overall mean PaO_2_, and the hyperoxia range were significantly associated with 28-day mortality (Table 3). After adjustment for age, hypertension, the indication for VA-ECMO, the SAPS II, and the arterial blood lactate on admission, we found that the mean daily peak PaO_2_, the absolute peak PaO_2_, the overall mean PaO_2_, and the hyperoxia range were independent predictors of 28-day mortality (OR [95%CI] per 10 mmHg increment: 2.65 [1.79–6.07], *P* = 0.02; 2.36 [1.67–4.82], *P* = 0.018; 2.85 [1.12–7.37], *P* = 0.028, respectively). Moderate and severe hyperoxia were found to be independent predictors of 28-day mortality. The predicted probability of 28-day mortality according to the mean daily peak PaO_2_, the absolute peak PaO_2_, and the overall mean PaO_2_ is presented in Additional file 2: Fig. S2.

**Table 3:** Association between hyperoxia and 28-day mortality, before and after adjustment

Variables	Unadjusted OR [95%CI] for a 10 mmHg increment	*P* value	Adjusted^a^ OR [95%CI] for a 10 mmHg increment	*P* value
Mean daily peak PaO_2_; *mmHg*^b^	2.77 [1.38–5.07]	0.005	2.65 [1.79–6.07]	0.02
Absolute peak PaO_2_; *mmHg*	2.48 [1.35–4.62]	0.004	2.36 [1.67–4.82]	0.018
*PaO*_*2*_* range; n (%)*				
200 mmHg	1 (reference)	–	1 (reference)	**–**
200–299 mmHg	1.88 [1.21–2.96]	0.005	1.82 [1.10–3.00]	0.02
≥ 300 mmHg	2.06 [1.00–4.27]	0.05	2.20 [1.00–5.31]	0.002
Overall mean PaO_2_; *mmHg* ^c^	2.66 [1.22–5.96]	0.015	2.85 [1.12–7.37]	0.028

OR**:** odds ratio. ^a^: ORs were obtained from a multivariate logistic regression with adjustment for age, hypertension, the indication for extracorporeal life support, the arterial blood lactate level on admission, and the Simplified Acute Physiology Score II. The ORs for the mean daily peak PaO_2_, absolute peak PaO_2_, and the mean PaO_2_ over 48 h were calculated for a 10-point increment in PaO_2_. ^b^: The mean daily peak PaO_2_ is the mean of the three daily peak PaO_2_ values (measured on admission (Day 0), Day 1, and Day 2). ^c^: The overall mean PaO_2_ is the mean of all PaO_2_ values measured between admission and Day 2

### Association between high level of oxygen and 28-day mortality

**Fig. 3:**
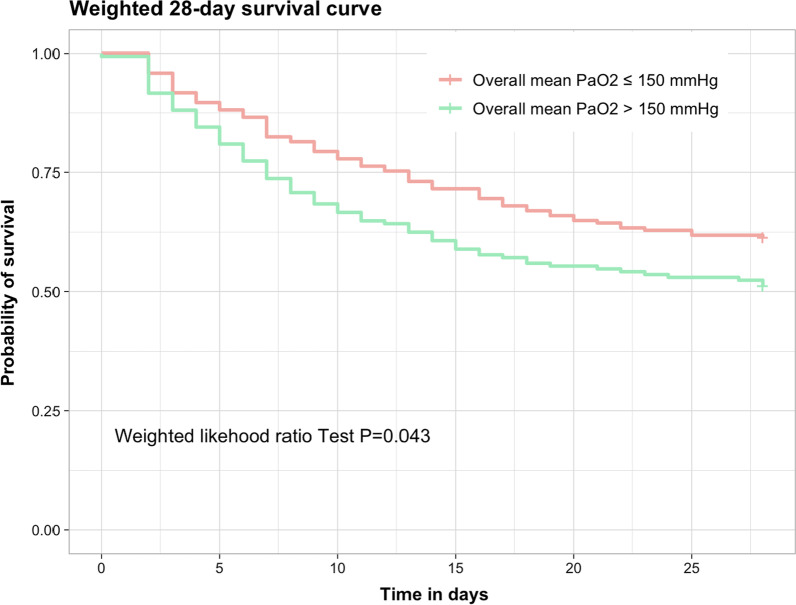
Propensity weighted Kaplan–Meier 28-day survival curve according to the overall mean PaO2 within the first 48 h after admission

Before weighting, a high level of oxygen (overall mean PaO_2_ > 150 mmHg) was significantly associated with 28-day mortality with an OR at 1.51 [1.03–2.23] (*P* = 0.035). After weighting, the association remained significantly different with an OR at 1.41 [1.01–2.08] (*P* = 0.041). Time to 28-day survival was significantly better in patients with an overall mean PaO_2_ ≤ 150 mmHg (Fig. 3).

### Survival analysis, by hyperoxia range

In a Kaplan–Meier analysis, mild hyperoxia was associated with greater 28-day survival, relative to moderate and severe hyperoxia (log-rank test: *P* = 0.0052) (Additional file 3: Fig. S3).

### Sensitivity analysis

Sixty-one patients had received femoro-axillary ECMO support. After the exclusion of these patients from the analysis, the OR for the association between oxygen parameters and 28-day mortality remained broadly consistent with the primary results (Additional file 4: Table S1).

## Discussion

Our findings suggest that exposure to hyperoxia during the early management of refractory CS is associated with a greater 28-day mortality rate. We also demonstrate a dose effect of the PaO_2_ increase on the risk of 28-day mortality, whatever the oxygen parameter assessed.

As mentioned above, published data on hyperoxia in patients on VA-ECMO are scarce. Hyperoxia seems frequent in the early management of VA-ECMO with almost 1 out of 2 patients concerned by the level of PaO_2_ over 300 mmHg [22]. However, the impact on outcomes is uncertain regarding the results of the two main studies with some contradictions: Al Kawaz et al. found a significant association between hyperoxia and mortality, whereas Munshi et al. did not [15], [13]. This disparity might have been due to differences in the study participants’ blood oxygen levels. Indeed, PaO_2_ was around 200 mmHg in Al Kawaz et al.’s study but only around 100 mmHg in Munshi et al*.*’s study. Our values were close to those reported by Al Kawaz *et al.*—confirming the risk of mortality at ~ 200 mmHg and over. Heterogeneity in the study population might be another explanatory factor. Munshi et al.’s study population was heterogeneous (including patients on venovenous ECMO or extracorporeal cardiopulmonary resuscitation, as well as patients on VA-ECMO for refractory CS (situations with marked differences in the mortality rate and pathophysiology), whereas Al Kawaz et al. focused on patients receiving VA-ECMO.

The duration of exposure to hyperoxia is probably also an important variable. Al Kawaz et al. showed that patients with hyperoxia (> 120 mmHg) for more than 50% of the time were significantly more likely to die [15]. We previously conducted a randomized controlled trial (RCT) of hyperoxia during cardiac surgery. Even though the PaO_2_ was over 400 mmHg in the intervention group with the inspired fraction oxygen at 1, the incidence of an adverse outcome was similar to that observed in the control group [23], 24. The hyperoxia exposure time was approximately 100 min. Hence, the area under the curve for PaO_2_ might be a more appropriate measure of the duration and level of exposure to hyperoxia. In the present study, we assessed the degree of hyperoxia during the first 48 h on ECMO support; our observation of a significant association with mortality suggests that even though very short exposures (a few hours) are harmless, the cumulative time interval for a harmful effect is less than 48 h. This finding highlights the importance of avoiding hyperoxia from the initiation of ECMO support onwards.

Although many large RCTs have investigated hyperoxia in mechanically ventilated or perioperative ICU patients, a significant association with mortality has not been found [25–27]. This raises the question of why hyperoxia would be harmful in patients on VA-ECMO support only. The initial severity of the patient’s status might have a role. One can hypothesize that ROS production is more pronounced in the most critical patients exposed to hyperoxia. In a large RCT of hyperoxia in septic shock patients (the HYPERS2 trial), a post hoc analysis revealed that the mortality rate was higher in patients with hyperoxia (FiO_2_ = 1) and an arterial blood lactate level > 2 mmol l^−1^. This finding confirms that the most critically ill patients were the most vulnerable to hyperoxia. In addition to the production of free radicals, hyperoxia can dysregulate the immune response and induce immunodepression [28]. All of our study participants (Table 1) met the criteria for the international Sepsis-3 definition of septic shock (with no evidence of infection), with mean arterial blood lactate of 6 mmol l^−1^ (> 2 mmol l^−1^) and a high SAPS II value [29].

Our study had some limitations. Firstly (and as with previous studies), the lack of consensus definitions of hyperoxia and a hyperoxia threshold was problematic. Secondly, oxygen demand might vary greatly as a function of various metabolic variables (body temperature, oxygen consumption, hemoglobin level, atmospheric pressure, blood pH, etc.). Overall, the definition of a single oxygen threshold appears not to be realistic, and oxygen demand variables should be taken into account (e.g., lactate clearance and central venous blood oxygen saturation). A third limitation relates to our inclusion of patients with femoro-axillary vs. femorofemoral VA-ECMO configurations [30]. The blood delivered by ECMO is usually fully saturated in oxygen, and retrograde flow will usually occur in the aorta. However, the aortic transition point between the anterograde flow produced to the patient’s heart and the retrograde flow produced by femorofemoral ECMO is difficult to determine [31, 32] Harlequin syndrome might result in differential hypoxia in the right arm and the brain, with poorly saturated blood delivered by ejection from the heart [33, 34] This is why we performed sensitivity analysis (Additional file 4: Table S1) after the exclusion of patients on femoro-axillary ECMO support. The association between hyperoxia and death (according to the OR) was consistent with our primary findings and so confirmed that the putative effect of hyperoxia was not influenced by the VA-ECMO configuration. Lastly, the retrospective design of the present study was associated with an inherent risk of bias. We are aware that other explicative variables are missing to describe the 28-day mortality in spite of the propensity analysis. Notably, the medical history before ECMO cannulation describing how severe was the patient (i.e., lactate course, vasoactive-inotropic score, cardiac fractional ejection) would have been valuable. The magnitude of the daily PaO_2_ is around 20 mmHg (Table 2) which seems a too narrow range to explain the difference in the mortality rate. Although we included patients with a refractory cardiogenic shock, we cannot exclude heterogeneity in the population study with different demands in oxygen. Recent analysis from large cohorts showed that the question of oxygen is complex. Low oxygen level might be beneficial for some pathologies, while higher oxygen level seems safer in sepsis [35–37]. Thus, that hypothetic heterogeneity in patients suggests that a unique oxygen level cannot be applied to all the patients but that an individualized oxygen is promoted based on the monitoring of oxygen consumption (central venous saturation, arterial lactate, etc.). A recent study reported the feasibility of oxygen challenge to assess lung ability to transport oxygen and could help in the individualization of oxygen settings [38]. The authors showed that good responders to oxygen challenge had a better risk of survival.

Overall, our findings suggest that a large RCT is now warranted. The BLENDER trial (ClinicalTrials.gov identifier NCT03841084) should provide some answers. Our present results (including the sensitivity analysis) might help to better interpret the BLENDER trial’s data. Study recruitment is ongoing, with a planned sample size of 300 patients allocated to standard care (SpO_2_ target 92–96%) or interventional care (FiO_2_ = 1). The main endpoint is the number of ICU-free days by post-randomization day 60.

## Conclusion

A high blood oxygen level during initial patient management with VA-ECMO was associated with a greater risk of 28-day mortality, in a dose-dependent manner. An ongoing RCT should provide answers on the management of oxygen levels during VA-ECMO.

## Supplementary Information

**Additional file 1.Fig. S1** : A Scatter plot examining the relationship between hemoglobin and maximal PaO2 at admission, with a fitted line representing the regression model and 95% confidence interval. B Scatter plot examining the relationship between ECMO flow rate and maximal PaO2 at admission, with a fitted line representing the regression model and 95% confidence interval.

**Additional file 2 Fig. S1**: Predicted probability of 28-day mortality according to mean daily peak PaO2 (A), absolute peak PaO2 (B), and overall mean PaO2 (C). Gray band indicates 95% confidence interval.

**Additional file 3 Fig. S1**: 28-day survival curve according to the first 48hrs admission hyperoxia range.

**Additional file 4. Table S1.** The association between hyperoxia and 28-day mortality after the exclusion of patients on femoro-axillary ECMO support, before and after adjustment.

## Data Availability

Available on request.

## Abbreviations

CS: Cardiogenic shock
ECMO: Extracorporeal membrane oxygenation
FiO_2_: Fraction of inspired oxygen
INTERMACS: Interagency registry for mechanically assisted circulatory support
IPTW: Inverse probability of treatment weighting
PaO_2_: Partial pressure of oxygen in arterial blood
SAPS II: Simplified Acute Physiology Score
SaO_2_: Arterial oxygen saturation
SCAI: Society for cardiovascular angiography and interventions
SpO_2_: Peripheral oxygen saturation
VA: Venoarterial
