# Supplementary figures and images for: Early hyperoxia and 28-day mortality in patients on venoarterial ECMO support for refractory cardiogenic shock: a bicenter retrospective propensity score-weighted analysis
Source: Crit Care. 2022 Aug 26;26:257. doi: 10.1186/s13054-022-04133-7 (PMC9414410; doi:10.1186/s13054-022-04133-7)

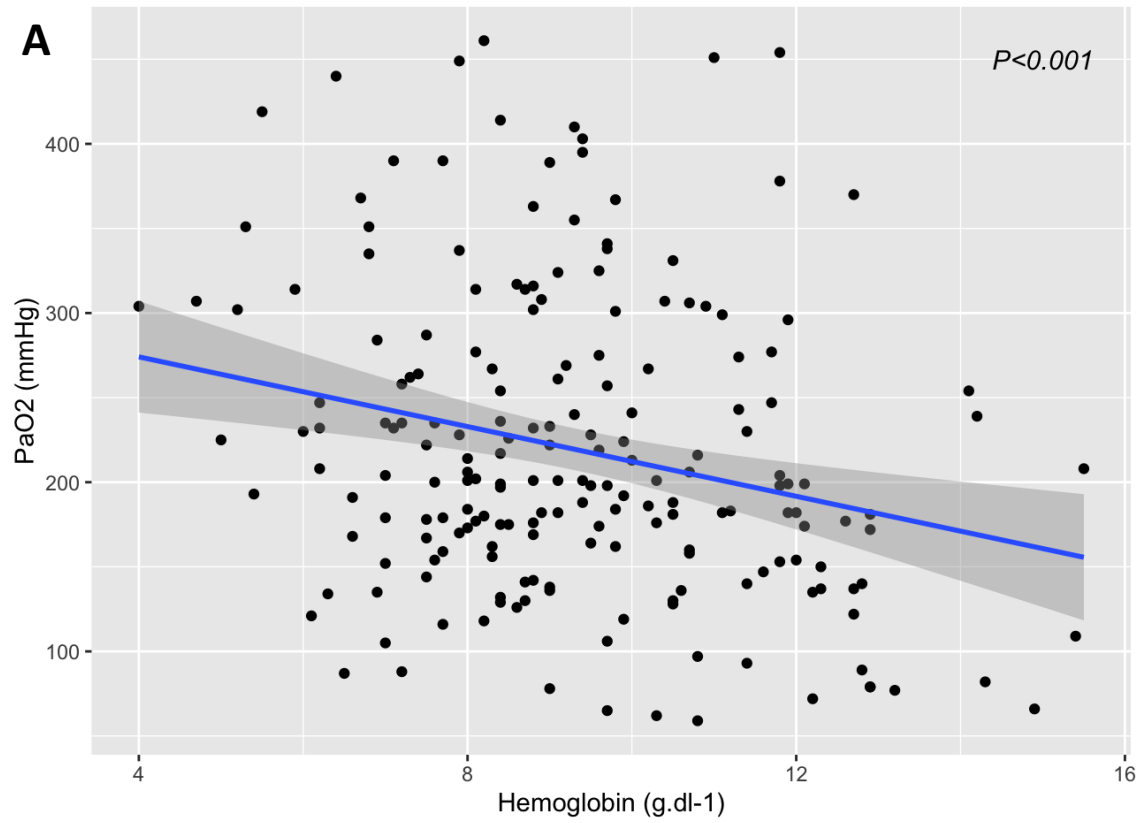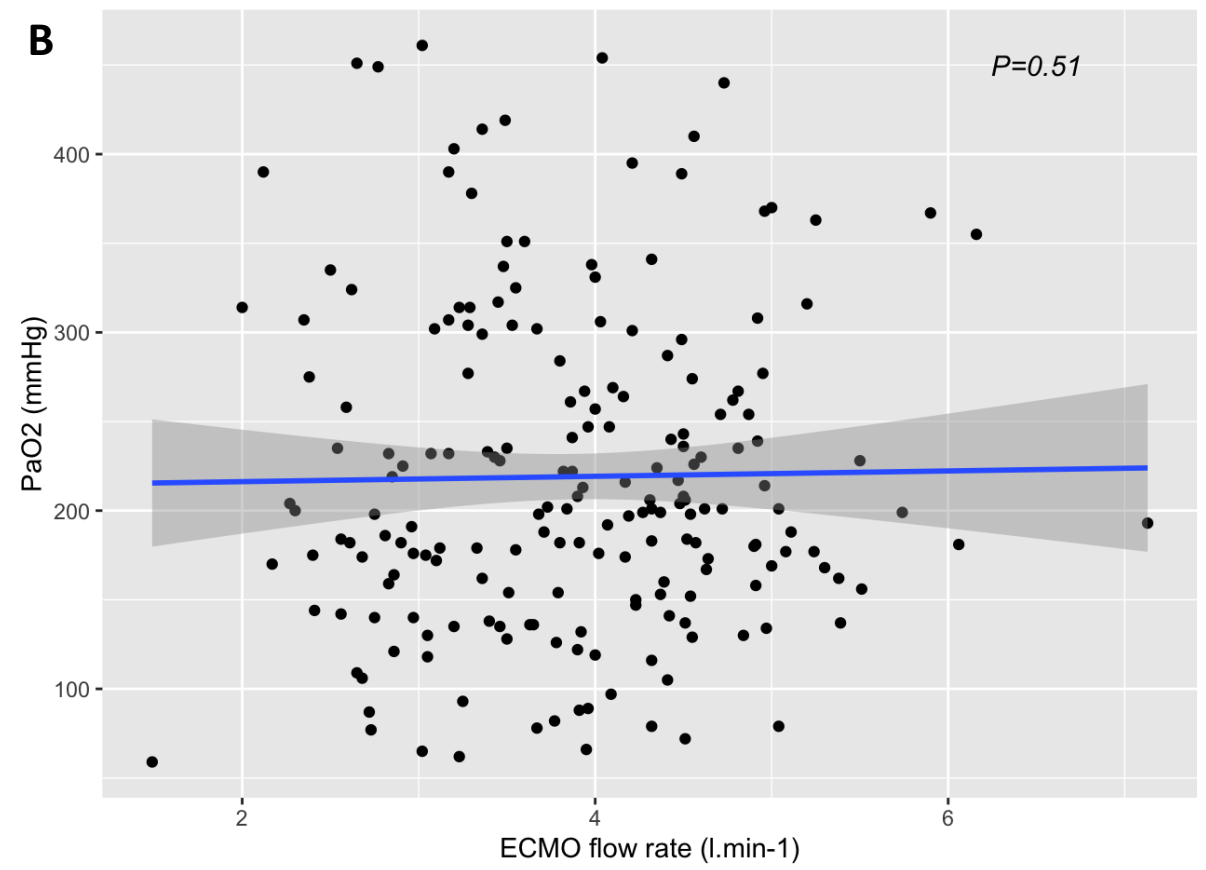

Supplement: Supplementary file 1 — Additional file 1.Fig. S1 : A Scatter plot examining the relationship between hemoglobin and maximal PaO2 at admission, with a fitted line representing the regression model and 95% confidence interval. B Scatter plot examining the relationship between ECMO flow rate and maximal PaO2 at admission, with a fitted line representing the regression model and 95% confidence interval. [file 13054_2022_4133_MOESM1_ESM.pdf]

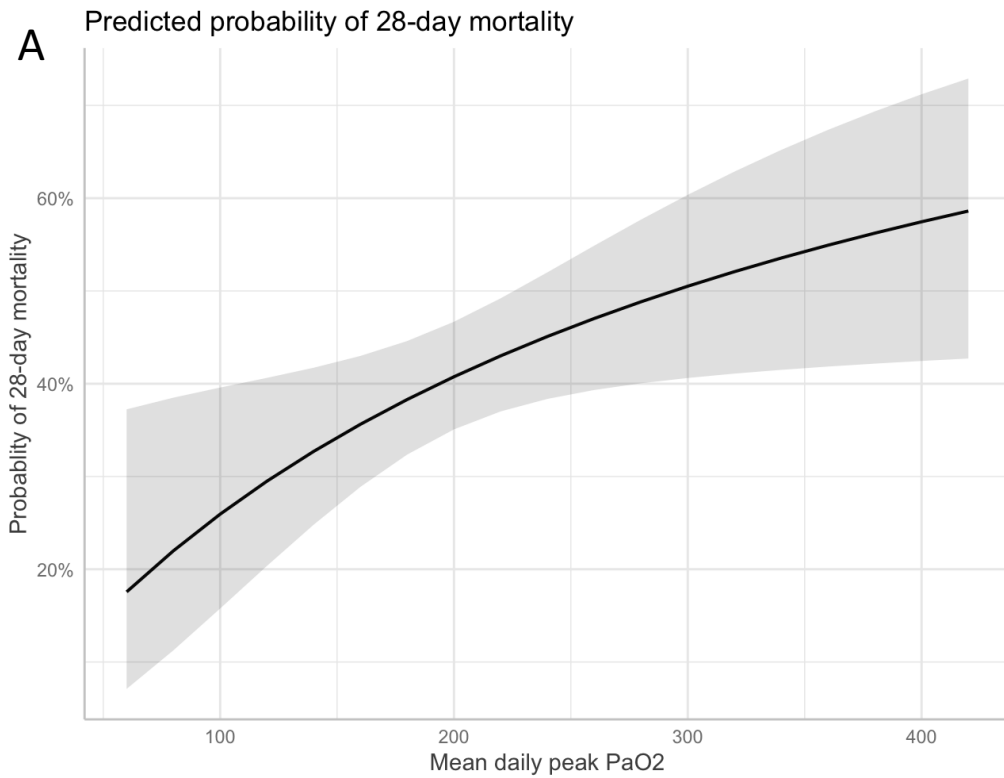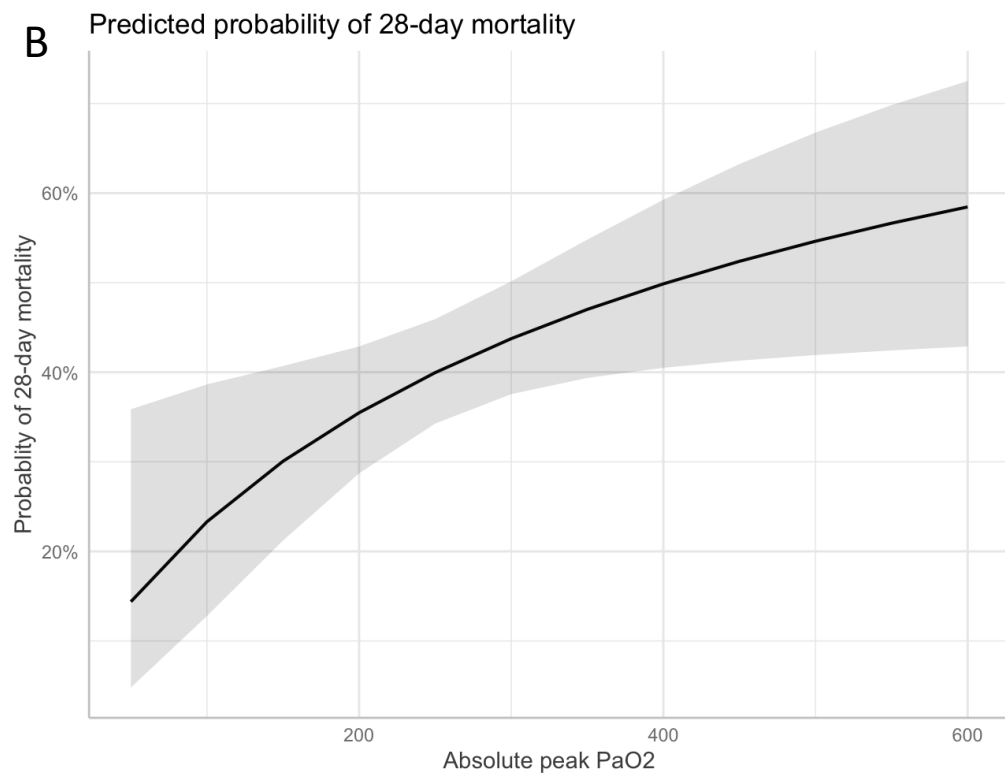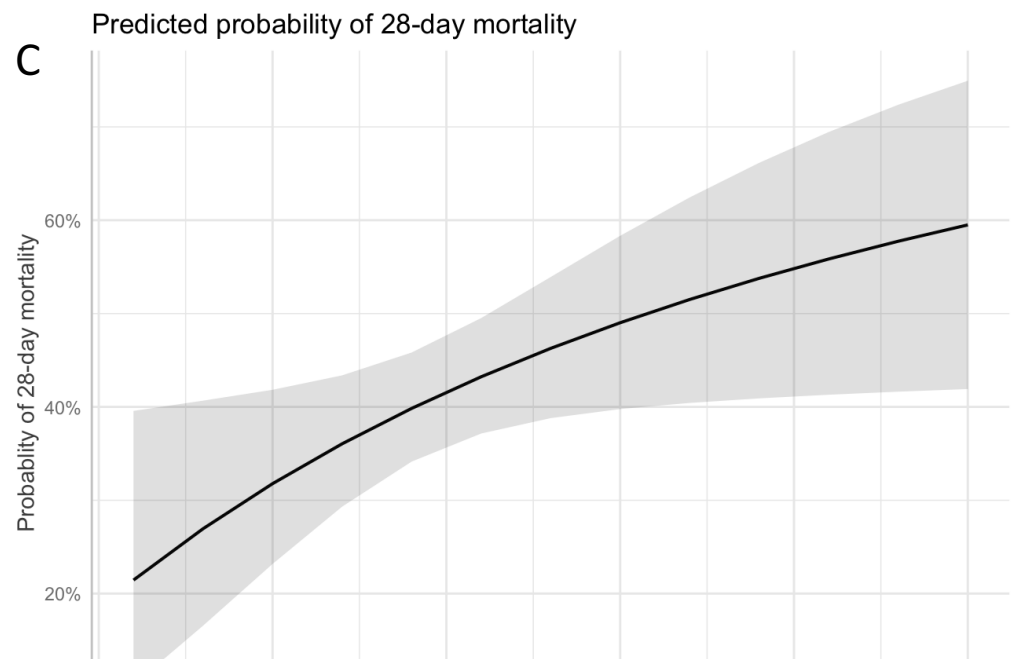

Supplement: Supplementary file 2 — Additional file 2 Fig. S1: Predicted probability of 28-day mortality according to mean daily peak PaO2 (A), absolute peak PaO2 (B), and overall mean PaO2 (C). Gray band indicates 95% confidence interval. [file 13054_2022_4133_MOESM2_ESM.pdf]

## 28-day survival curve

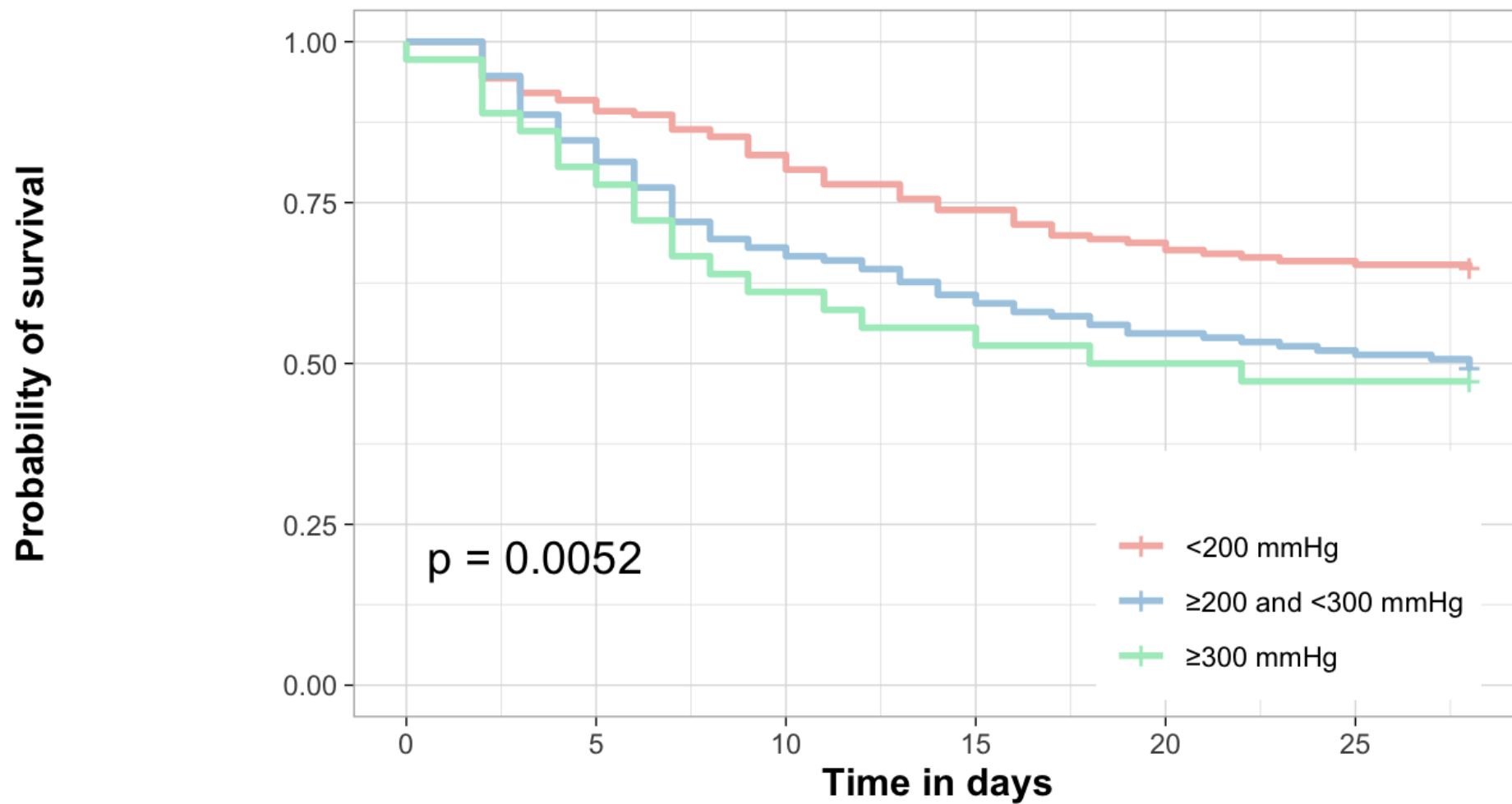

## Number at risk

|                    |              |     |     |     |     |     |
|--------------------|--------------|-----|-----|-----|-----|-----|
| <200 mmHg          | 176          | 160 | 145 | 130 | 121 | 116 |
| ≥200 and <300 mmHg | 150          | 127 | 102 | 91  | 82  | 78  |
| ≥300 mmHg          | 36           | 29  | 22  | 20  | 18  | 17  |
|                    | 0            | 5   | 10  | 15  | 20  | 25  |
|                    | Time in days |     |     |     |     |     |

Supplement: Supplementary file 3 — Additional file 3 Fig. S1: 28-day survival curve according to the first 48hrs admission hyperoxia range. [file 13054_2022_4133_MOESM3_ESM.pdf]
