# Supplementary material for: Early hyperoxia and 28-day mortality in patients on venoarterial ECMO support for refractory cardiogenic shock: a bicenter retrospective propensity score-weighted analysis
Source: Crit Care. 2022 Aug 26;26:257. doi: 10.1186/s13054-022-04133-7 (PMC9414410; doi:10.1186/s13054-022-04133-7)
Supplement: Supplementary file 4 — Additional file 4. Table S1. The association between hyperoxia and 28-day mortality after the exclusion of patients on femoro-axillary ECMO support, before and after adjustment. [file 13054_2022_4133_MOESM4_ESM.docx]

| **Variable** | **Unadjusted**  **OR [95%CI] for a 10-mmHg increment** | ***P*-value** | **Adjusted ^a^**  **OR [95%CI] for a 10-mmHg increment** | ***P*-value** |
| --- | --- | --- | --- | --- |
| Mean daily peak PaO_2_; *mmHg* ^b^ | 3.06 [1.43-6.70] | 0.004 | 2.63 [1.08-6.56] | 0.03 |
| Absolute peak PaO_2_; *mmHg* | 2.52 [1.30-4.97] | 0.007 | 2.46 [1.04-4.94] | 0.04 |
| PaO_2_ range; *n (%)* |  |  |  |  |
| 200 mmHg | 1 (reference) | - | 1 (reference) | **-** |
| 200 – 299 mmHg | 1.86 [1.15-3.02] | 0.012 | 1.76 [1.02-3.06] | 0.04 |
| ≥300 mmHg | 2.38 [1.11-5.15] | 0.026 | 2.61 [1.01-6.96] | 0.05 |
| Mean PaO_2_ over 48 h; *mmHg* ^c^ | 3.16 [1.34-7.61] | 0.009 | 3.03 [1.09-8.69] | 0.04 |

**Supplementary table. The association between hyperoxia and 28-day mortality after the exclusion of patients on femoro-axillary ECMO support, before and after adjustment.**

OR**:** odds ratio. ^a^: ORs were obtained from a multivariate logistic regression with adjustment for age, hypertension, the indication for extracorporeal life support, the arterial blood lactate level on admission, and the Simplified Acute Physiology Score II. The ORs for the mean daily peak PaO_2_, absolute peak PaO_2_ and the mean PaO_2_ over 48 h were calculated for a 10-point increment in PaO_2_. ^b^: The mean daily peak PaO_2_ is the mean of the three-daily peak PaO_2_ values (measured on admission (Day 0), Day 1 and Day 2). ^c^: The mean PaO_2_ over 48 h is the mean of all PaO_2_ values measured between admission and Day 2.
